# Supplementary material for: siRNA Treatment Enhances Collagen Fiber Formation in Tissue-Engineered Meniscus via Transient Inhibition of Aggrecan Production
Source: Bioengineering (Basel). 2024 Dec 23;11(12):1308. doi: 10.3390/bioengineering11121308 (PMC11727199; doi:10.3390/bioengineering11121308)
Supplement: Supplementary file 1 [file bioengineering-11-01308-s001.zip › Lopez et al Supplemental Figure Captions.pdf]

Supplemental Figure Captions

Supplemental Figure S1: A) Disc construct punched to 8 mm diameter 2 mm height composed of soft collagen-gel. B) Meniscus construct with linear geometry comprised of two bone plugs on either edge (outside of red dashed lines) and a soft collagen-based gel in between. These constructs are clamped at the bone plugs to provide biomechanical stimulation for improved fiber formation.

Supplemental Figure S2: Transfection efficiency (%) of siRNA transfected fibrochondrocytes, as determined by comparison of phase contrast and fluorescence images of siGLO Red transfected fibrochondrocytes at day 3 of culture.

Supplemental Figure S3: DNA content in 3D disc constructs at 30 days of culture (n=3) of untransfected control, siGLO Red control, and siACAN constructs. Analyzed using 1-way ANOVA with Tukey's multiple comparisons test.

Supplemental Figure S4: Strain histograms at 15% applied strain obtained via MATLAB's NCORR analysis of confocal elastography images from untransfected controls, Lipofectamine controls, siGLO Red controls, and siACAN constructs. Two Gaussian curves were fitted to the histograms, as indicated by the two dashed lines showing each component overlayed on the graphs.

Supplemental Figure S5: Proportion of data under each Gaussian curve fitted to strain histograms from untransfected control, Lipofectamine control, siGLO Red control, and siACAN constructs at 15% applied strain.

Supplemental Figure S6: A) 2D cultures of Control, Lipofectamine control, and siACAN cells stained with calcein AM and ethidium homodimer (Invitrogen LIVE/DEAD™ Viability/Cytotoxicity Kit) and imaged on a ZeissLSM880 inverted confocal microscope using manufacturer-recommended excitation and emission spectra (calcein AM (488 nm/537 nm), ethidium homodimer (561 nm/661 nm) to visualize live (left) and dead (right) cells at days 3 and 6 of culture. Scale bars = 100 µm B) Quantification of cell viability in 2D monolayer culture of control (black circles), Lipofectamine control (red triangles), and siACAN (pink triangles) at days 3 and 6.
