# Supplementary figures and images for: siRNA Treatment Enhances Collagen Fiber Formation in Tissue-Engineered Meniscus via Transient Inhibition of Aggrecan Production
Source: Bioengineering (Basel). 2024 Dec 23;11(12):1308. doi: 10.3390/bioengineering11121308 (PMC11727199; doi:10.3390/bioengineering11121308)

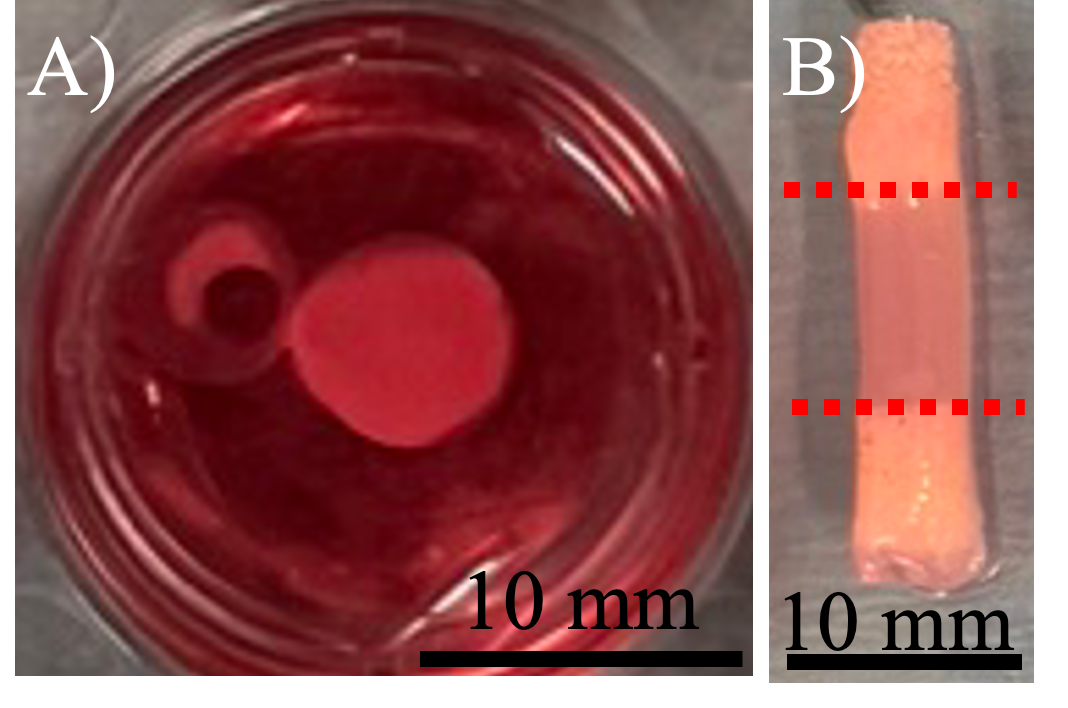

Supplement: Supplementary file 1 [file bioengineering-11-01308-s001.zip › SIFigure1.tif]

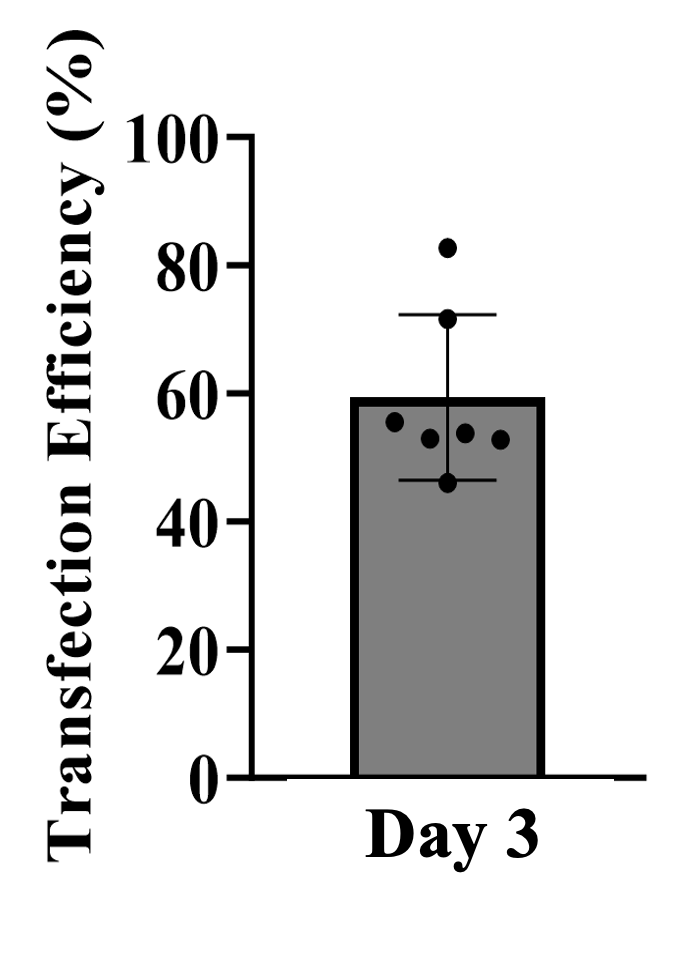

Supplement: Supplementary file 1 [file bioengineering-11-01308-s001.zip › SIFigure2.tif]

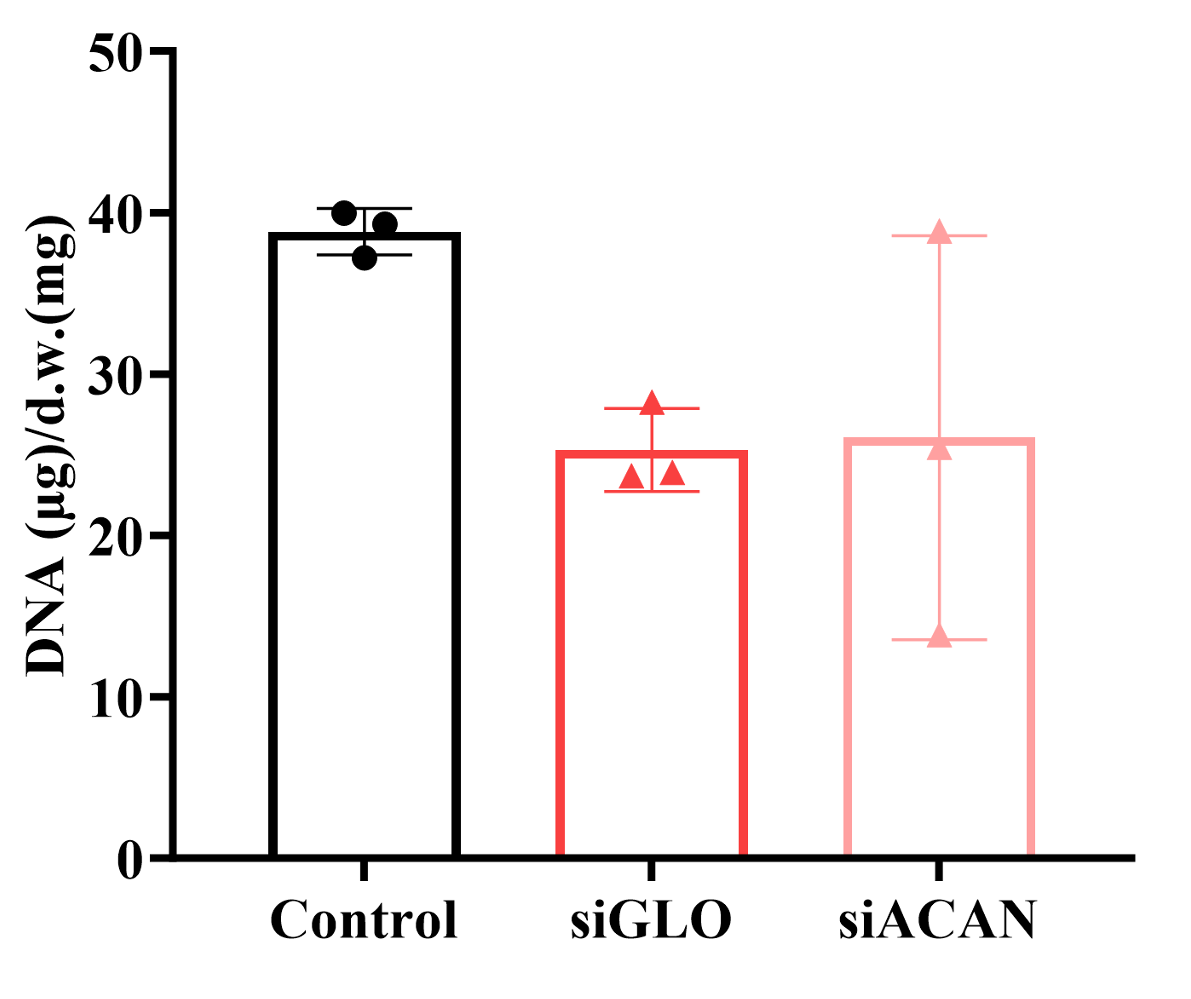

Supplement: Supplementary file 1 [file bioengineering-11-01308-s001.zip › SIFigure3.tif]

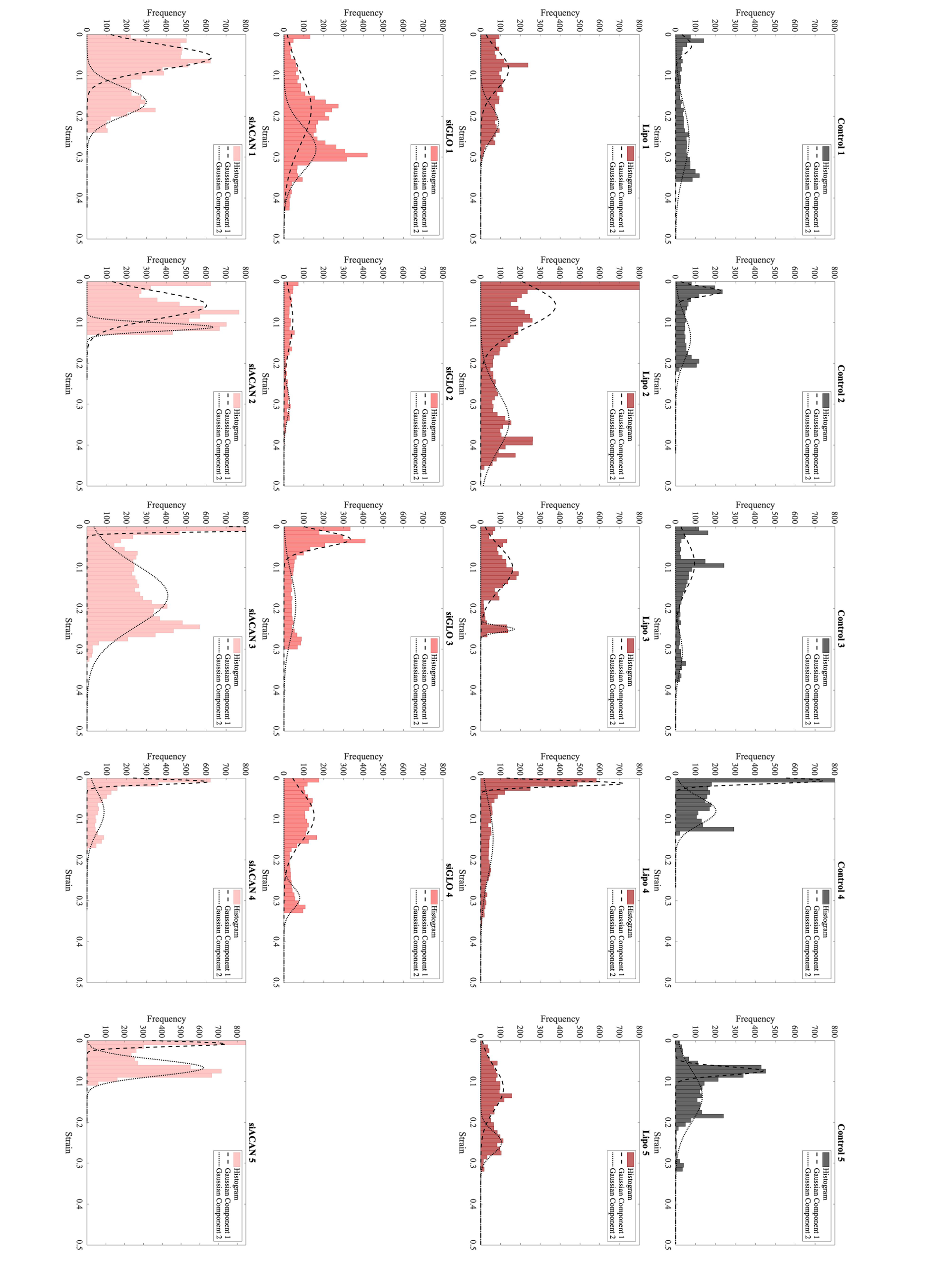

Supplement: Supplementary file 1 [file bioengineering-11-01308-s001.zip › SIFigure4.tiff]

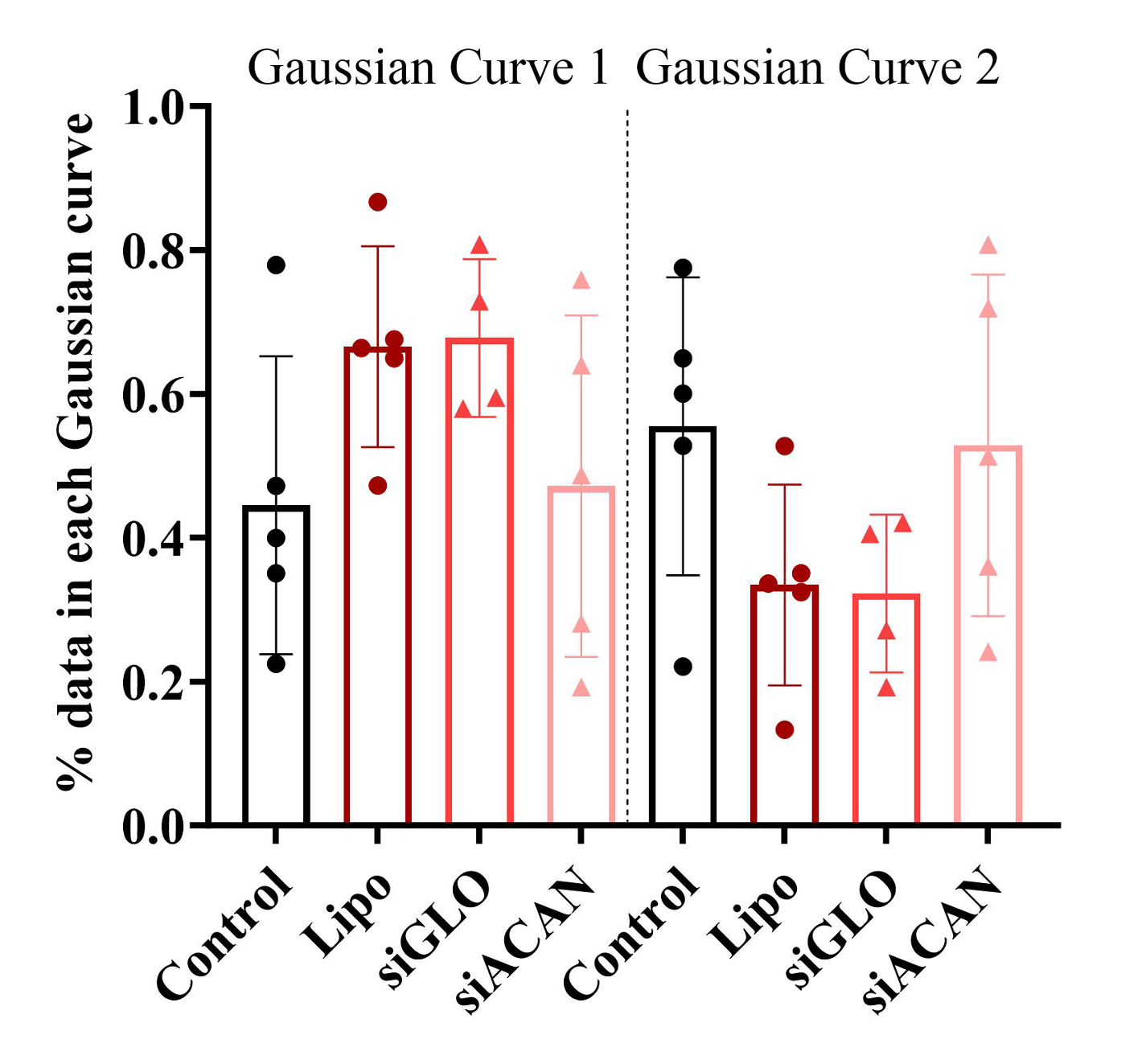

Supplement: Supplementary file 1 [file bioengineering-11-01308-s001.zip › SIFigure5.tif]

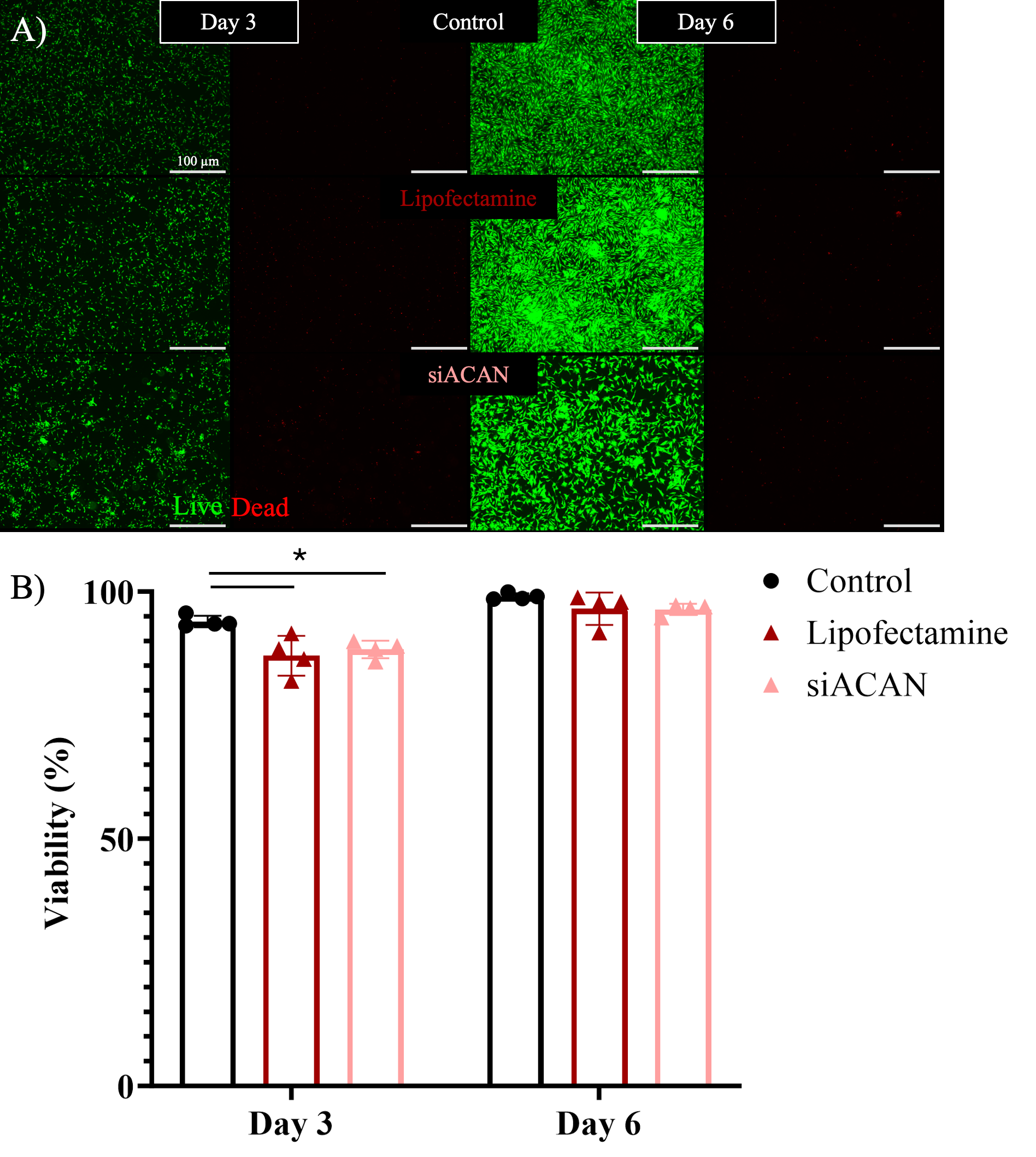

Supplement: Supplementary file 1 [file bioengineering-11-01308-s001.zip › SIFigure6.tif]
